# Fluoxetine Counteracts the Cognitive and Cellular Effects of 5-Fluorouracil in the Rat Hippocampus by a Mechanism of Prevention Rather than Recovery

**DOI:** 10.1371/journal.pone.0030010

**Published:** 2012-01-17

**Authors:** Laura Lyons, Maha ELBeltagy, Geoffrey Bennett, Peter Wigmore

**Affiliations:** 1 School of Biomedical Sciences, University of Nottingham, Nottingham, United Kingdom; 2 Department of Anatomy, Menoufiya University, Shibin el Kom, Egypt; Case Western Reserve University, United States of America

## Abstract

5-Fluorouracil (5-FU) is a cytostatic drug associated with chemotherapy-induced cognitive impairments that many cancer patients experience after treatment. Previous work in rodents has shown that 5-FU reduces hippocampal cell proliferation, a possible mechanism for the observed cognitive impairment, and that both effects can be reversed by co-administration of the antidepressant, fluoxetine. In the present study we investigate the optimum time for administration of fluoxetine to reverse or prevent the cognitive and cellular effects of 5-FU.

Male Lister-hooded rats received 5 injections of 5-FU (25 mg/kg, i.p.) over 2 weeks. Some rats were co-administered with fluoxetine (10 mg/kg/day, in drinking water) for 3 weeks before and during (preventative) or after (recovery) 5-FU treatment or both time periods (throughout). Spatial memory was tested using the novel location recognition (NLR) test and proliferation and survival of hippocampal cells was quantified using immunohistochemistry. 5-FU-treated rats showed cognitive impairment in the NLR task and a reduction in cell proliferation and survival in the subgranular zone of the dentate gyrus, compared to saline treated controls. These impairments were still seen for rats administered fluoxetine after 5-FU treatment, but were not present when fluoxetine was administered both before and during 5-FU treatment. The results demonstrate that fluoxetine is able to prevent but not reverse the cognitive and cellular effects of 5-FU. This provides information on the mechanism by which fluoxetine acts to protect against 5-FU and indicates when it would be beneficial to administer the antidepressant to cancer patients.

## Introduction

Adjuvant chemotherapy is commonly used to treat patients with breast cancer; however it is associated with many unwanted side effects. One such effect is cognitive impairment, which can encompass lack of concentration, problems with memory formation and general confusion [1] and has been reported to last for up to several years after completion of chemotherapy treatment [2], [3]. With the increasing survival of cancer sufferers, it is becoming important to understand the causes of chemotherapy induced cognitive impairment and to find ways to prevent it and improve patient quality of life.

The antimetabolite, 5-fluorouracil (5-FU), is commonly used in combination with other agents to treat cancer and has been associated with cognitive impairment in patients [4], [5]. Its ability to cross the blood-brain barrier by passive diffusion enables it to affect the brain when given systemically [6]. A small number of studies, performed in rodents, have previously examined the effects of 5-FU, with the majority finding that the drug impaired cognition and suppressed hippocampal cell proliferation [6].

Consequently, the cytotoxic effect of chemotherapy on the proliferation of neural stem and precursor cells required for adult hippocampal neurogenesis has been considered as a possible mechanism for chemotherapy-induced cognitive impairment [7], [8], [9], [10]. The subgranular zone of the dentate gyrus is one of a limited number of regions where neurogenesis persists throughout adulthood [11]. Memory formation and spatial memory are both functions of the hippocampus and the proliferation and integration of the neuronal precursors into existing circuits is thought to play a functional role in this process [12], [13].

Fluoxetine, a selective serotonin reuptake inhibitor (SSRI) antidepressant, has been shown to increase cell proliferation in the hippocampus in both rodents [14], [15] and humans [16] and improve memory in patients with impaired cognition [17], [18], [19]. Furthermore, recent rodent investigations in our group showed that fluoxetine can reverse the impaired spatial memory and reduced proliferation of hippocampal cells caused by treatment with 5-FU [7] and methotrexate [10] chemotherapy.

In the present study, we utilised a rat model to confirm that co-administration of fluoxetine during 5-FU treatment counteracts the cognitive deficits and the reduction in proliferation and survival of cells found in the subgranular zone caused by the chemotherapy. The novel location recognition (NLR) task was used to assess spatial working memory after 5-FU and fluoxetine treatment. Cells which were proliferating in the dentate gyrus at the end of the experiment were quantified by Ki67, a protein expressed in all stages of the cell cycle [20]. To investigate the effect of 5-FU and fluoxetine on the survival of newly generated cells in the SGZ, Bromodeoxyuridine (BrdU) was injected over the 3 days immediately before 5-FU treatment. The number of surviving cells, marked at this time, was quantified at the end of the experiment.

**Figure 1:**
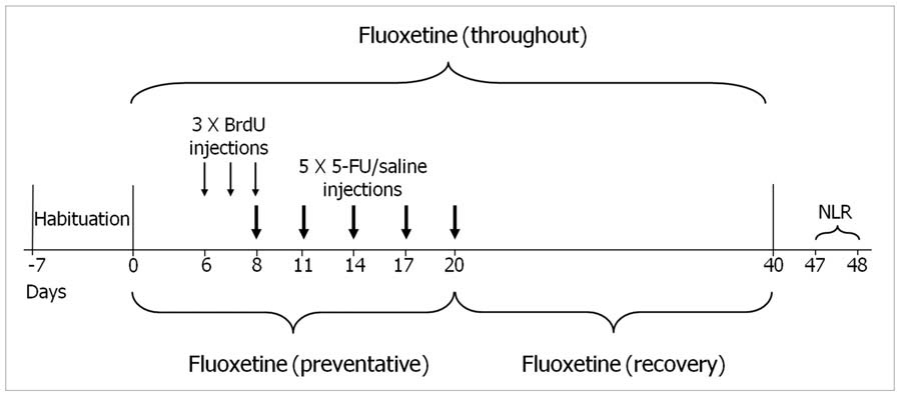
Time line showing protocol of drug administration and behavioural testing. Arrows represent single i.p. injections of BrdU (fine) and 5-FU/saline (thick). Brackets represent the period of time for which fluoxetine was administered in the drinking water. The day after Novel location recognition (NLR) behavioural testing, animals were killed and their brains were removed.

To understand whether the mode of action of fluoxetine prevents or recovers the cognitive decline and reduced neurogenesis caused by 5-FU, fluoxetine was given for 3 different time periods; before and during (preventative), after (recovery), and for both of these periods combined (throughout), the 5-FU treatment period (Fig. 1). These studies have demonstrated for the first time that the action of the antidepressant fluoxetine, in chemotherapy treatment, prevents the 5-FU induced cognitive deficits and cellular changes but has little effect in recovery.

## Materials and Methods

### Ethics statement

Principles of laboratory animal care in this study were in accordance to UK Home Office Guidance regulations, within the “moderate” severity band, with approval from the University of Nottingham ethical committee board under permit number 40/3283. Throughout the experiment, discomfort to animals was kept to an absolute minimum. Animals remained in good health throughout the study and never dropped more than 10% of their highest body weight.

### Animals and treatment

Male Lister-hooded rats (175–200 g; Charles River, UK) were randomly allocated to vehicle, 5-FU, fluoxetine, 5-FU+fluoxetine (throughout), 5-FU+fluoxetine (preventative) or 5-FU+fluoxetine (recovery) groups (each, n = 12). Animals were housed in cages of four and allowed to habituate for 1 week prior to drug administration.

Rats were administered 5-FU (25 mg/kg, 5 i.p. doses, each 3 days apart, at a volume of 2.5 ml/kg, dissolved in 0.9% sterile saline; Medac, Germany) or 0.9% sterile saline at an equivocal volume. Fluoxetine treated animals initially had a lower fluid intake than controls, probably due to a temporary taste aversion to fluoxetine [21]. However, by the end of the experiment, all groups had the same fluid intake. 3 BrdU injections were administered to all groups, 24 h apart starting 2 days prior to their first 5-FU/saline injection (100 mg/kg, i.p., at a volume of 4 ml/kg; Sigma Aldrich, UK). This method of administration was selected for maximum incorporation of BrdU into the nuclei of the cells [22].

Mean water consumption and mean animal weights per cage were determined every 3 days to estimate a dose of 10 mg/kg/day of fluoxetine (Pinewood Healthcare, Ireland, oral solution) administered in the drinking water, for fluoxetine treated groups of rats [10]. This mode of drug administration was seen to be advantageous as it avoided the possible stress of isolation rearing, administration by gavage or repeated injection and has been used by a number of groups [23], [24], [25]. In addition, clinical reports have suggested that studies of chemotherapy induced cognitive impairment in patients can be confounded by stress [26]. Thompson et al. have shown that rats treated with fluoxetine in drinking water for 37 days have fluoxetine and norfluoxetine serum levels of 281±44 and 1209±123 nmol/l respectively [27], levels comparable to levels achieved by injection [28]. The period of administration for fluoxetine was at least 20 days which is sufficient to have anxiolytic effects [29] and increase hippocampal neurogenesis [15] in rats. Behavioural testing was carried out a week after termination of fluoxetine treatment, as fluoxetine and its primary metabolite norfluoxetine have a long half life and take 3 days to wash out of the system [28].

Drinking water treated with fluoxetine was administered to the 5-FU+fluoxetine (preventative) group starting 5 days before the first BrdU injection and to the 5-FU+fluoxetine (recovery) group starting the day of the last 5-FU/saline injection, both administrations were for 20 days. The fluoxetine and the 5-FU+fluoxetine (throughout) groups received fluoxetine for the whole period of the experiment, a total of 40 days (Fig. 1).

Throughout the experiment, rats were maintained under a 12 h light/dark cycle (7.00/19.00h), food and water was provided *ad libitum* and weighed every 3 days or daily during 5-FU administration. Fluoxetine dose was calculated from mean animal weight and fluid consumption per cage. All procedures were in accordance to UK Home Office Guidance regulations and with local ethical committee approval.

### Behavioural testing

#### Novel location recognition (NLR)

The NLR two-trial spatial memory task is a spatial variant of a two trial object recognition task adapted from Dix and Aggleton [30]. It was carried out 1 week after fluoxetine treatment ended, as described by Lyons et al [10]. In brief, rats were habituated to an arena (49 width×66 length×40 height cm) for 30 min, 24 h prior to testing (during which their mean velocity was measured using EthoVision 4.1) and again for 3 min, 5 min before the familiarisation trial. In the 3 min familiarisation trial, rats were placed in the arena to explore two identical objects (weighted water bottles) in different locations. Rats were removed for a 15 min retention period and then reintroduced to the arena for the 3 min choice trial in which one object had been moved to a different location. Exploration time of both objects in both trials was recorded blind three times and averaged using a stopwatch from digitised recordings. Experiments were conducted at an illumination of 80 Lux between 9.00 and 14.00 h.

#### Brain tissue preparation

Rats were killed by rapid stunning and cervical dislocation the day after behavioural testing. Brains were removed, cut sagittally and cryopreserved in 30% sucrose solution for three hours at 4°C, then submerged in OCT-compound (VWR International Ltd, UK) and snap frozen in liquid nitrogen-cooled isopentane. Brains were stored at −80°C until being sectioned along the coronal plane using a Leica CM 100 cryostat (Leica Microsystems, UK) at 20 µm thickness at −20°C. The sections were thaw mounted onto 3-aminopropylmethoxysaline (APES)-coated slides and stored at −20°C until used for immunohistochemistry.

#### Immunohistochemistry

For immunostaining a systematic random sampling technique was used [31]. Every 20^th^ section throughout the entire length of the dentate gyrus was selected, resulting in a total of 9–11 sections per brain. All immunohistochemistry incubations were carried out at room temperature in a light-proof humidity chamber.

Ki67 and BrdU staining was carried out as described by Lyons et al. [10]. Briefly, sections were incubated with monoclonal mouse Ki67 primary antibody (1∶300; Vector laboratories, UK) for 1 h, followed by 1 h incubation with Alexa 555 donkey anti-mouse (1∶300; Invitrogen, UK) or with polyclonal sheep BrdU primary antibody (1∶100; Abcam, UK) for 16–20 h followed by Alexa 488 donkey anti-sheep secondary antibody (1∶300; Invitrogen, UK) in PBS. Sections were mounted with (diamidinophenylindole) DAPI (1.5 µg/ml) nuclear marker (Vector laboratories, UK) and coverslipped.

All staining was viewed and quantified at ×40 on a Nikon EFD-3 fluorescence microscope. BrdU and Ki67 positive cells which co-localised with the DAPI nuclear staining within the subgranular zone of both hippocampal blades were counted. By combining cell counts per section for the whole dentate gyrus and multiplying by 20, an estimate of total immuno positive cell numbers was produced [32]. All counting was performed blind.

#### Statistical analysis

Student's paired *t* tests were used to compare exploration times of animals in the familiarisation and choice trials. Preference indices (PI) were created by expressing time spent exploring the object in the novel location as a percentage of the sum of exploration time of novel and familiar locations in the choice trial, to create a single value to compare between groups [33]. PI was compared to 50% chance using a one-sample *t* test. Two-way repeated measured ANOVA was run to determine difference in animal weight and fluid intake between treatment groups. One-way ANOVA was used to compare total exploration time and average velocity of the animals and cells counts. When ANOVA was significant it was followed by Bonferroni post-hoc test. Statistical analysis and graphs were created using GraphPad Prism 5 and significance was regarded as *p*<0.05.

## Results

### 5-FU and fluoxetine reduce weight gain and fluid intake

**Figure 2:**
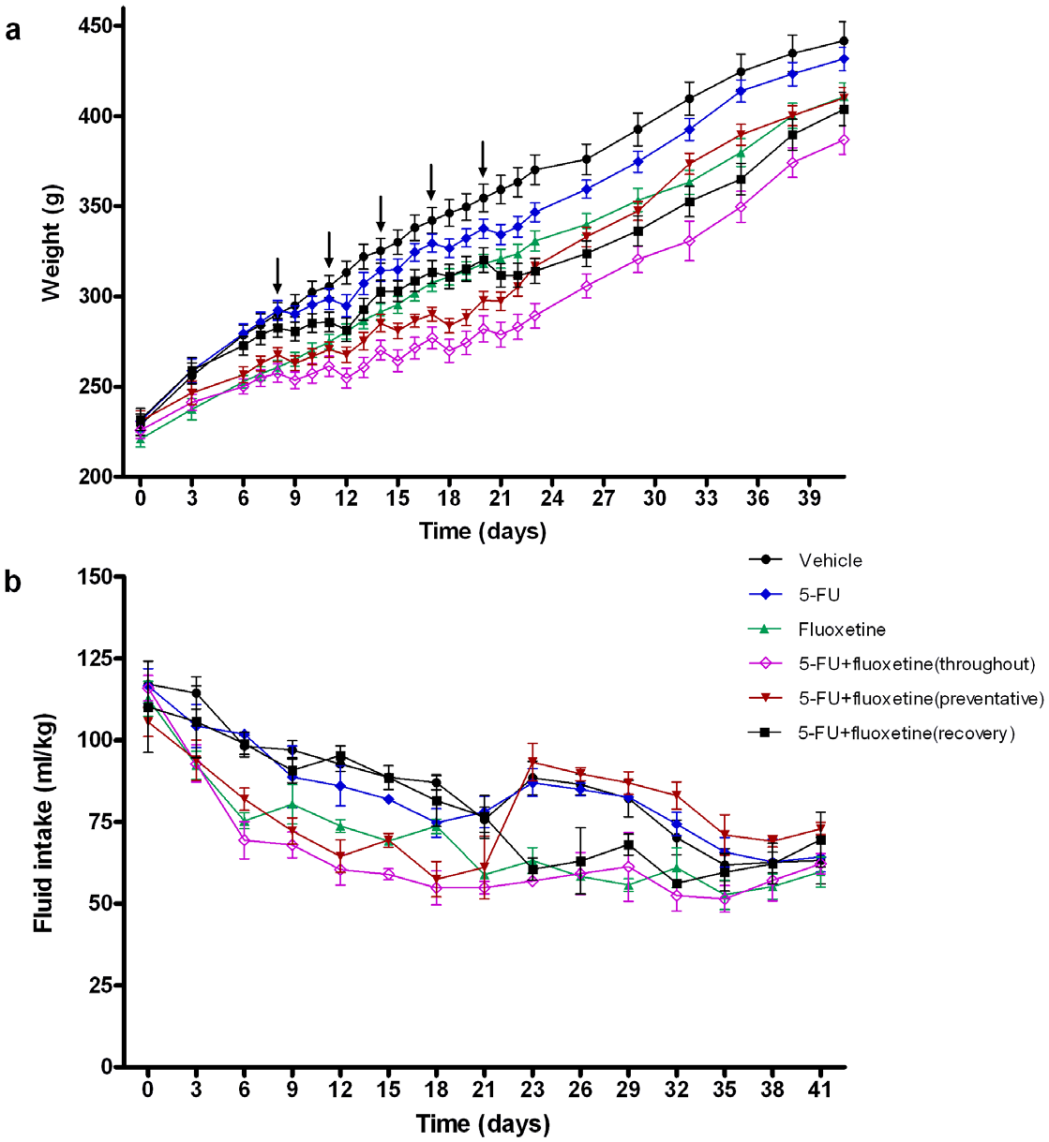
Body weights of rats (a) and their fluid intake (b) during fluoxetine treatment period (mean ± SEM). Arrows indicate 5-FU (20 mg/kg)/saline injections. Fluoxetine was given in drinking water (10 mg/kg/day) from day 1 to day 20 for the 5-FU+fluoxetine (preventative) group, from day 21 to day 40 for the 5-FU+fluoxetine (recovery) group and from day 0 to day 40 for the 5-FU+fluoxetine (throughout) and the fluoxetine alone groups.

Both treatment and time had a significant effect on body weight (*F*_5,1848_ = 11.50, *F*_28,1848_ = 2040, *p*<0.001 respectively, two-way repeated measures ANOVA, Fig. 2a). Both 5-FU and fluoxetine significantly reduced weight gain which is attributed to disruption of intestinal absorption by 5-FU [34] and fluoxetine [35]. Treatment and time significantly affected the amount of water drunk (*F*_5,169_ = 17.93, *F*_14,168_ = 52.09, *p*<0.001 respectively, two-way repeated measures ANOVA, Fig. 2b). However, by the end of the experiment no significant difference was seen (*p*>0.05, one-way ANOVA). Although, the fluid intake fell per kg as animals increase in weight, the actual fluid intake did not drop per animal (data not shown).

### Fluoxetine reverses the behavioural deficits caused by 5-FU when administered in prevention but not recovery

**Figure 3:**
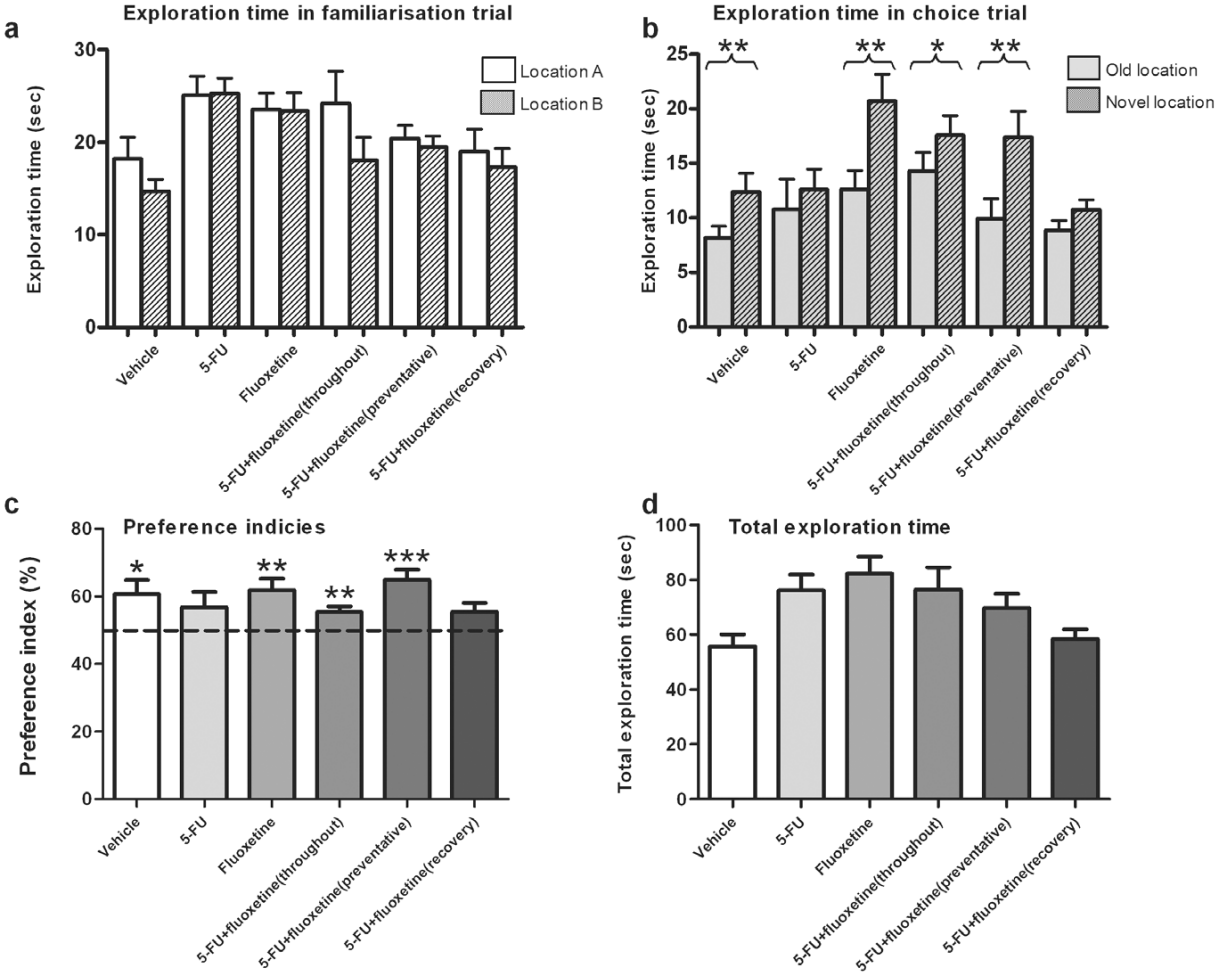
Mean exploration times (mean ± SEM) of the rats for each object in the familiarisation (a) and choice (b) trials. There was no significant difference in exploration time of either object for any group in the familiarisation trial (*p*>0.05). In the choice trial, all groups spent significantly longer exploring the object in the novel location (*p<0.05, **p<0.01), except the groups receiving 5-FU alone or 5-FU with fluoxetine in recovery (*p*>0.05). Preference indices (PI, (c), mean ± SEM) were created by expressing time spent exploring the object in the novel location as a percentage of the sum of exploration time of novel and familiar locations in the choice trial (Bruel-Jungerman et al. 2005). All groups were significantly different from chance (*p<0.05, **p<0.01, ***p<0.001), other than the groups receiving 5-FU alone or 5-FU with fluoxetine in recovery (*p*>0.05). The total exploration time (mean ± SEM) for both trial combined (d) did not differ significantly between groups (*p*>0.05).

The NLR test makes use of the preference of rats for novelty to measure the ability of rats to discriminate between objects in novel and familiar locations. In the familiarisation trial, the rats explored 2 identical objects and no group showed a significant difference in exploration time for either object (*p*>0.05, Fig. 3a) indicating no preference for either object's location. During the choice trial (Fig. 3b) however, the groups of vehicle treated rats, rats receiving only fluoxetine and rats receiving both 5-FU and fluoxetine throughout or for prevention were able to perform the memory task, spending significantly longer exploring the object in the novel location compared to the object in the familiar location (all *p*<0.05). In contrast, rats treated with 5-FU only or 5-FU with fluoxetine in recovery showed no object preference, and no significant difference in exploration time for either object (*p*>0.05), indicating an impairment in memory. Conversion of raw exploration times showed that the means of the PI of these 2 groups did not differ from a level of 50% chance, whereas the mean PI of the other groups was significantly different (Fig. 3c).

**Figure 4:**
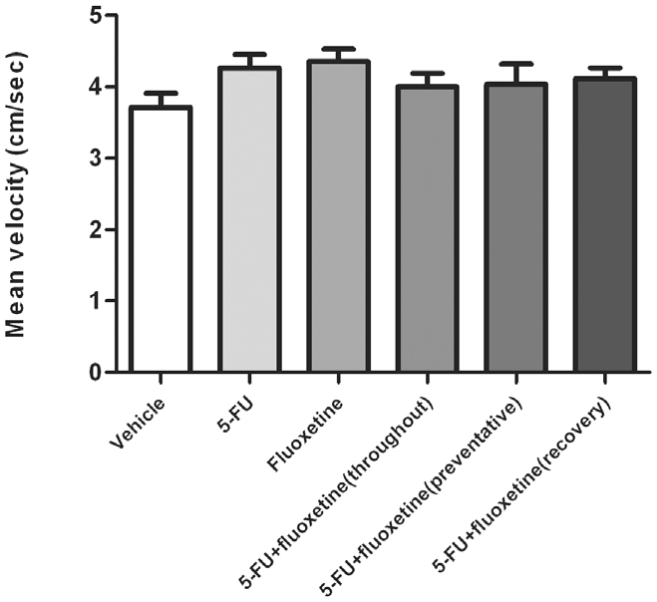
Mean velocity (mean ± SEM) of rats recorded during the habituation period using EthoVision 4.1. No significant difference (*p*>0.05) was found between each group.

No significant difference was found between groups for either total exploration time (Fig. 3d) for both trials or mean velocity (Fig. 4) indicating none of the groups were impaired in their activity.

### Fluoxetine abolishes the reduction in cell proliferation in the dentate gyrus caused by 5-FU when administered in prevention but not recovery

**Figure 5:**
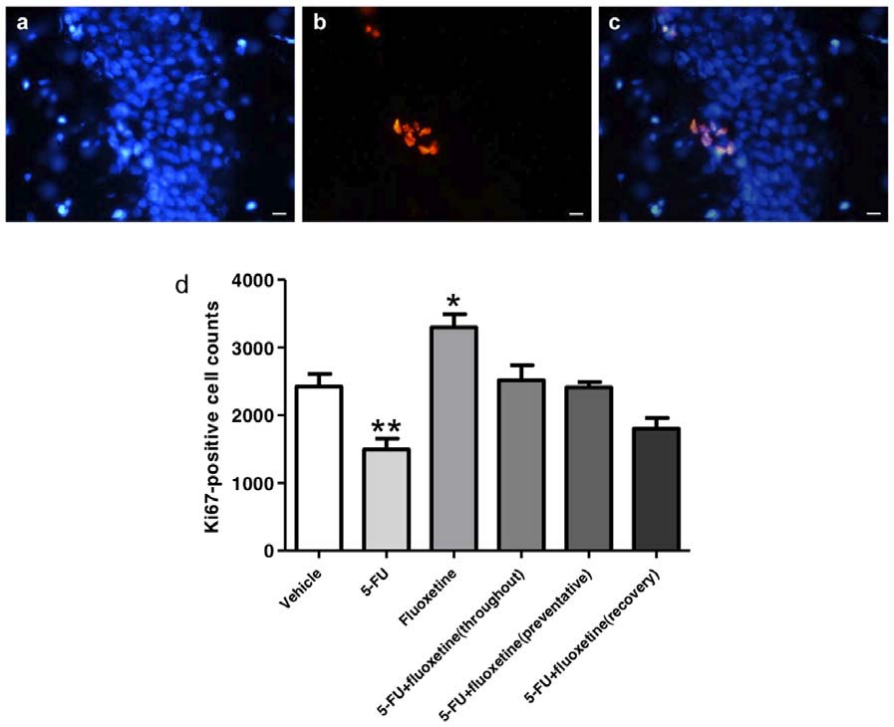
Photographs of the nuclei of cells in the SGZ of the dentate gyrus (blue, a), Ki67-positive cells (red, b) and the photos merged (c). Bar scales indicate 20 µm. Total numbers of Ki67-positive cells (mean ± SEM) in the dentate gyrus were estimated from cell counts (d). Rats receiving 5-FU had significantly fewer Ki67-positive cells (*p*<0.01) in the subgranular zone (SGZ) and rats receiving fluoxetine had significantly more (*p*<0.05) than the saline-treated control group. The other treatment groups receiving both 5-FU and fluoxetine did not significantly differ from the controls (*p*>0.05).

Cell proliferation in the subgranular zone at the end of the experiment (30 days after the final saline/5-FU injection) was quantified using Ki67 (Fig. 5a, b, c, d). Rats receiving only 5-FU had a significantly lower number of Ki67-positive cells and rats receiving only fluoxetine had a significantly larger number compared to vehicle-treated controls. The number of Ki67-positive cells in rats treated with both 5-FU and fluoxetine did not significantly differ from the controls when fluoxetine was administered throughout; in prevention and in recovery. However rats administered fluoxetine in recovery had the lowest numbers of Ki-67 positive cells. These results indicate that 5-FU has a long term effect, reducing cell proliferation in the subgranular zone for at least four weeks. This effect can be counteracted by fluoxetine if it is administered before and during the 5-FU treatment period, but only incompletely counteracted if fluoxetine is administered after chemotherapy treatment.

### Fluoxetine prevents the reduction in cell survival caused by 5-FU when administered during but not after chemotherapy

**Figure 6:**
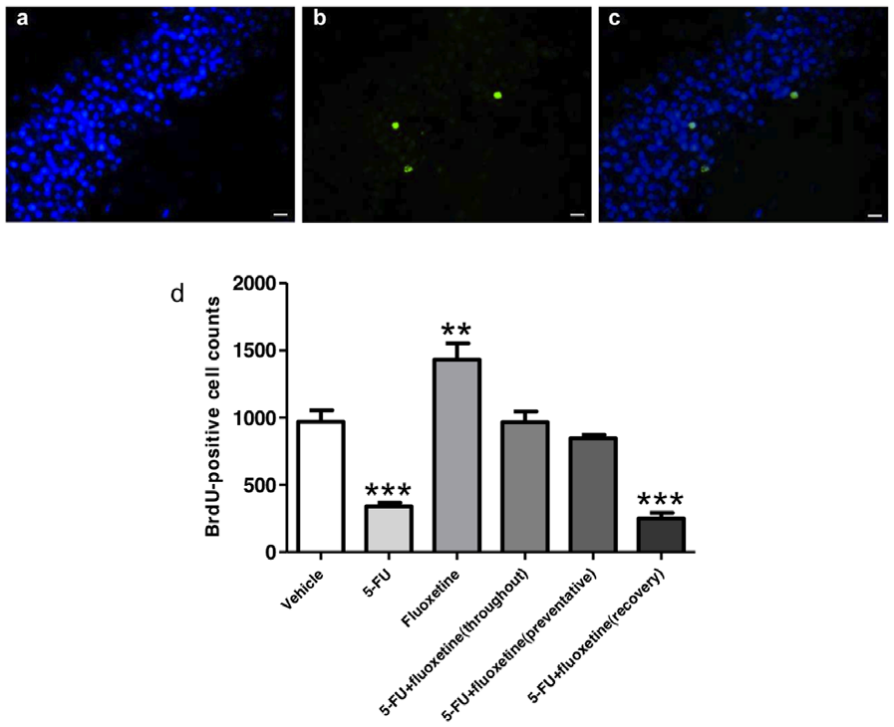
Photographs of the nuclei of cells in the dentate gyrus (blue, a), BrdU-positive cells (green, b) and the photos merged (c). Bar scales indicate 20 µm. Total numbers of BrdU-positive cells (mean ± SEM) in the dentate gyrus were estimated from cell counts (d). Rats receiving 5-FU had significantly fewer BrdU-positive cells (*p*<0.001) in the SGZ and rats receiving fluoxetine had significantly more (*p*<0.01) than the saline-treated control group. Treatment groups receiving both 5-FU and fluoxetine throughout and in prevention did not significantly differ from the controls (*p*>0.05). The group receiving 5-FU with fluoxetine only in recovery had significantly fewer BrdU-postive cells than the control group (*p*<0.001).

A course of 3 BrdU injections was given to animals preceding 5-FU or saline injection to label cells dividing at the start of 5-FU treatment. BrdU-positive cells were counted in the dentate gyrus and SGZ at the end of the experiment to quantify the survival of these cells (Fig. 6a, b, c, d). There were significantly more BrdU-positive cells in rats treated with fluoxetine only compared with the control group and significantly fewer positive cells in rats treated with 5-FU. The rats treated with both 5-FU and fluoxetine did not have a significantly different number of BrdU-positive cells than the controls when fluoxetine was administered throughout and in prevention, but when fluoxetine was only administered in recovery, the rats had a significantly smaller number. These results suggest that when administered before and during, but not after 5-FU treatment, fluoxetine can protect neural precursors from cell loss induced by 5-FU.

## Discussion

The present study showed that the chemotherapy agent 5-FU caused a memory impairment which was associated with a reduction in both the proliferation and survival of neural precursors in the subgranular zone of the dentate gyrus. These effects were counteracted when the SSRI antidepressant fluoxetine was co-administered before and during (preventative) but not after (recovery) 5-FU treatment.

The NLR test was chosen as a test of spatial memory as it is hippocampal dependent [36] and relies on the animals' spontaneous preference for novelty, and does not require positive or negative reinforcers. In the present study, 5-FU-treated animals were unable to recognise an object in a novel as opposed to a familiar location, suggesting a memory deficit in contrast to saline treated controls. This is in line with patient reports of chemotherapy-induced cognitive impairments. Fluoxetine has been previously shown to have cognitive benefits for rats treated with chemotherapy when given before during and after chemotherapy treatment [10]. This positive effect remained when fluoxetine was given before and during the 5-FU treatment but was not found when fluoxetine administration started after the final 5-FU injection, indicating that fluoxetine can protect from the effects of chemotherapy but cannot compensate after chemotherapy has been given.

Disruption of neurogenesis is a possible mechanism by which 5-FU causes cognitive impairment [7], [8]. Production and survival of new neurones in the hippocampus is thought to be essential for spatial memory and learning [12], [13] and cognitive impairments are seen when neurogenesis is disrupted by irradiation [37], [38], chemotherapy drugs [7], [10], [39] or genetic manipulation [40]. In the present study the effects of 5-FU and fluoxetine on production and survival of new hippocampal cells were examined. 5-FU treatment on its own significantly reduced the survival of cells BrdU labelled at the start of 5-FU treatment (Fig. 6). In addition quantification of cell division (Ki-67 positive cells) at the end of the experiment, 30 days after completion of 5-FU treatment, showed that cell proliferation was significantly less than controls (Fig. 5). The effect of fluoxetine on animals not receiving chemotherapy was to increase cell survival and cell proliferation (Fig. 5; 6) as previously reported [10]. Fluoxetine given either only during 5-FU treatment (preventative) or throughout the whole experiment (throughout) showed no reduction in cell proliferation or cell survival at the end of the experiment. Animals administered fluoxetine for 20 days after the end of 5-FU treatment, showed the same reduction in cell survival as the chemotherapy only group indicating that loss of cells which were dividing at the start of 5-FU treatment, occurred during chemotherapy treatment and was not affected by subsequent fluoxetine treatment. The level of cell proliferation in this group, at the end of the experiment, was not significantly different from controls but lay between the values found in control and 5-FU only treated groups (Fig. 5). This may indicate some recovery in cell proliferation with fluoxetine administration after 5-FU treatment.

The results in the present study are the first to examine the time course of the effects of fluoxetine on the response to the chemotherapy agent, 5-FU, and are consistent with the earlier work from our group [7], [8] as well as other studies which show that 5-FU affects memory immediately (2 days) [41] and for at least 5 weeks [42] after drug administration as well as a reduction in cell proliferation in the SGZ [7], [8]. The proliferation, survival and apoptosis of neural progenitors are all involved in memory formation and the stage of growth of newly-born neurones when learning and memory takes place is an important factor [40]. In the present study, memory was affected 4 weeks after 5-FU chemotherapy treatment indicating that 5-FU had a long lasting effect. We show here that fluoxetine increases the survival of cells by protecting newly forming neurones from 5-FU.

Fluoxetine may be acting by increasing the level of brain-derived neurotrophic factor (BDNF) [43], [44] [45] and indirectly affecting neurogenesis or by directly increasing the proliferation of hippocampal neural stem cells [46]. Several recent reports have shown that fluoxetine itself has anti cancer properties [47], [48], [49] and can potentiate the action of some chemotherapy agents [50]. Further work will be needed to establish the actions of fluoxetine on different cancers and its interaction with chemotherapy agents in particular as fluoxetine can inhibit the cytochrome P450 enzymes involved in drug metabolism [51].

The results of the present study show that fluoxetine can protect newly born hippocampal neurones from the cytotoxic effects of 5-FU. If similar effects in preventing chemotherapy-induced memory deficits are found in patients, these results may offer a relatively simple way to counteract cognitive impairment in this situation.
